# Identification of Preferred DNA-Binding Sites for the *Thermus thermophilus* Transcriptional Regulator SbtR by the Combinatorial Approach REPSA

**DOI:** 10.1371/journal.pone.0159408

**Published:** 2016-07-18

**Authors:** Michael W. Van Dyke, Matthew D. Beyer, Emily Clay, Kamir J. Hiam, Jonathan L. McMurry, Ying Xie

**Affiliations:** 1 Department of Chemistry and Biochemistry, Kennesaw State University, Kennesaw, Georgia, United States of America; 2 Department of Molecular and Cellular Biology, Kennesaw State University, Kennesaw, Georgia, United States of America; 3 Department of Computer Science, Kennesaw State University, Kennesaw, Georgia, United States of America; Florida International University Bimolecular Sciences Institute, UNITED STATES

## Abstract

One of the first steps towards elucidating the biological function of a putative transcriptional regulator is to ascertain its preferred DNA-binding sequences. This may be rapidly and effectively achieved through the application of a combinatorial approach, one involving very large numbers of randomized oligonucleotides and reiterative selection and amplification steps to enrich for high-affinity nucleic acid-binding sequences. Previously, we had developed the novel combinatorial approach Restriction Endonuclease Protection, Selection and Amplification (REPSA), which relies not on the physical separation of ligand-nucleic acid complexes but instead selects on the basis of ligand-dependent inhibition of enzymatic template inactivation, specifically cleavage by type IIS restriction endonucleases. Thus, no prior knowledge of the ligand is required for REPSA, making it more amenable for discovery purposes. Here we describe using REPSA, massively parallel sequencing, and bioinformatics to identify the preferred DNA-binding sites for the transcriptional regulator SbtR, encoded by the *TTHA0167* gene from the model extreme thermophile *Thermus thermophilus* HB8. From the resulting position weight matrix, we can identify multiple operons potentially regulated by SbtR and postulate a biological role for this protein in regulating extracellular transport processes. Our study provides a proof-of-concept for the application of REPSA for the identification of preferred DNA-binding sites for orphan transcriptional regulators and a first step towards determining their possible biological roles.

## Introduction

Genome projects have yielded considerable information since the sequencing of the first whole microorganism genome, *Haemophilus influenza*, in 1995 [1,2]. Of prime significance is the identification of open reading frames (ORFs), which can encode for potentially important proteins. In certain organisms for which a great deal of genetic and biochemical information is available, *e*.*g*., model microorganisms such as *Escherichia coli* and *Saccharomyces cerevisiae*, some level of understanding exists regarding the biological functions of most of their proteins [3–5]. However, considerable gaps in our knowledge persist even in these organisms, with many ORFs encoding predicted “orphan” proteins for which little to no information is available [6,7]. A poignant example is recent synthetic biology attempts at generating a minimal bacterial genome. The latest effort, containing only 473 quasi-essential genes from *Mycoplasma mycoides*, still possesses 149 ORFs encoding proteins with unknown biological functions [8]. Orphan proteins become even more problematic in less well-studied organisms, where most of our understanding of potential proteins stem from their sequence homology with presumed counterparts in better-studied organisms [9,10]. Thus for both well studied and less well-characterized organisms, there remains much to be accomplished before their genome products and their biological roles are fully understood.

*Thermus thermophilus* HB8 is an extremely thermophilic, aerobic eubacteria [11]. Given its ease in culturing and relatively small genome (2.12 Mb, 2245 genes), it has become a model organism for genetic manipulation, structural genomics, and systems biology [12–15]. Presently ongoing research is being undertaken to systematically determine the three-dimensional structure of all *T*. *thermophilus* HB8 proteins as part of the Structural and Functional Whole Cell Project, with the goal of achieving a comprehensive mechanistic understanding of all its biological phenomena at an atomic level [16]. To date, many of its genes have been cloned and its proteins overexpressed, with over 466 crystal structures determined (http://www.thermus.org/e_index.htm). However, while a gene analysis of the *T*. *thermophilus* HB8 genome has been able to ascribe possible functions for proteins encoded by 1360 of 2226 genes, the others remain hypothetical proteins with unknown functions. Such limits our complete understanding of systems biology in this model organism.

Transcription factors are proteins that control the expression of genes by regulating the process of RNA synthesis, otherwise known as transcription. One distinctive characteristic of these proteins is their recognition of specific DNA sequences, often located in a region proximal to the start site of transcription known as the promoter. In most organisms, genes encoding transcription factors are quite plentiful, constituting ~5% of all protein-coding genes [17,18]. This reflects the fact that transcription is the primary means of regulating gene expression and that most organisms need to respond to a variety of changes in their environment necessitating a tight level of control over the expression of specific sets of genes. For a well-characterized organism such as *E*. *coli*, 271 of its 4140 identified protein-coding genes are postulated to encode for transcription factors [19]. Of these, detailed DNA binding information (*e*.*g*., position-specific scoring matrices or sequence logos) is available for less than half [20]. This is even more apparent for a less well-characterized organism such as *T*. *thermophilus*, where of its 2173 identified protein-coding genes, only ~70 are predicted to be transcription factors and detailed DNA binding information is only available for a handful [21–30]. A complete knowledge of its transcription factors and the genes they control will be instrumental in furthering our understanding of regulatory networks present in this model organism.

**Fig 1:**
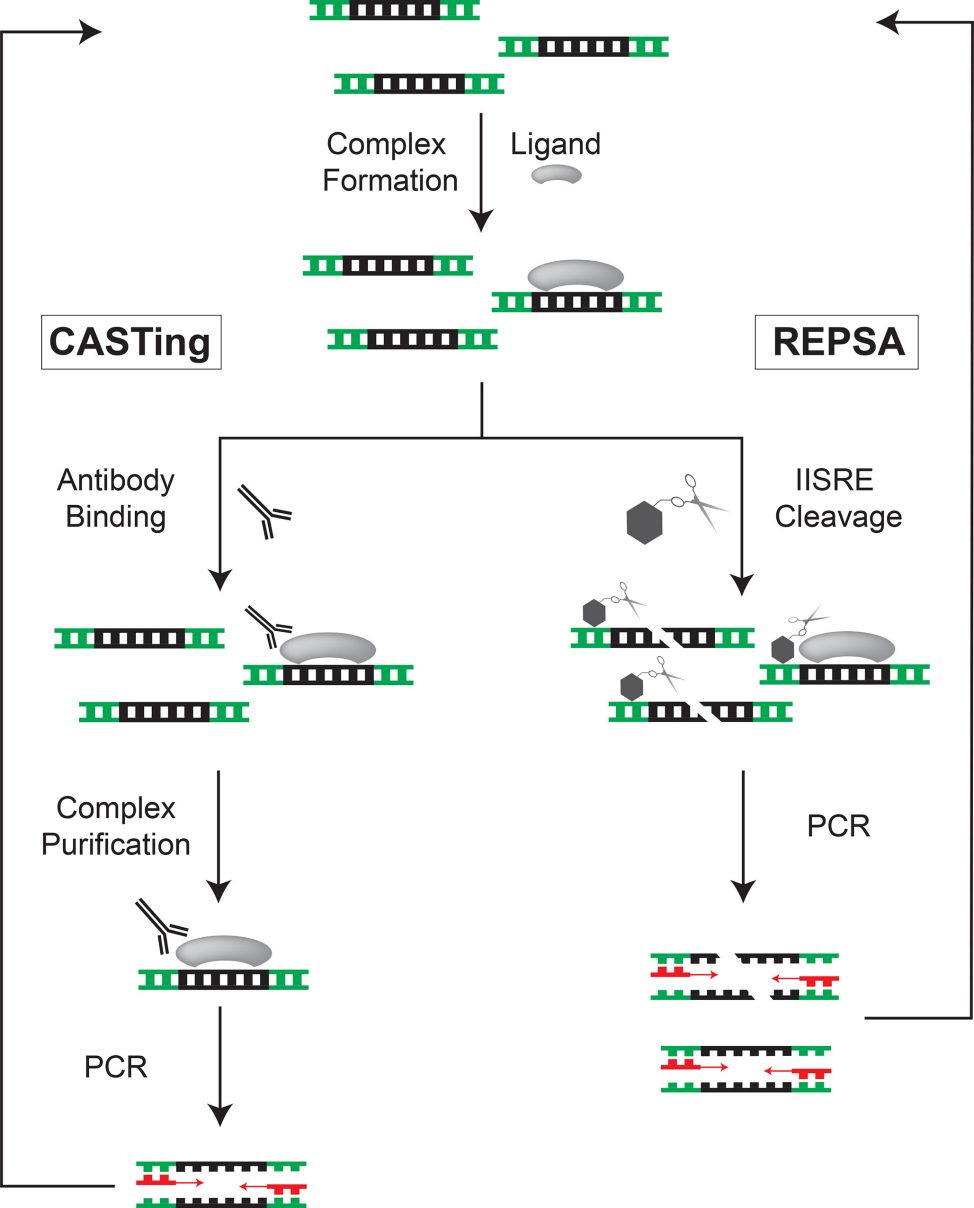
Comparison of CASTing and REPSA selection methods. Shown are flow diagrams depicting Cyclic Amplification and Selection of Targets (CASTing) and Restriction Endonuclease Protection Selection and Amplification (REPSA), combinatorial selection methods for the identification of preferred ligand-binding sequences in duplex DNA. Both methods rely on large populations of randomized DNA sequences, ligand-binding to a subpopulation of these DNAs, and PCR amplification of selected DNAs. However, CASTing and similar methods rely on the physical separation of ligand-bound from unbound DNAs (*e*.*g*., immunoprecipitation) for its selection process, whereas REPSA utilizes ligand-dependent interference with a template inactivation process (type IIS restriction endonuclease cleavage) for selection.

The intrinsic specificity of DNA recognition by transcription factors provides a unique aspect that allows for a rapid means of obtaining first insights into their potential biological roles. Numerous technologies exist for exploring the DNA-binding specificity of proteins. Foremost among these are combinatorial selection methods, including CASTing, SELEX and SAAB, which use large populations of randomized sequence oligonucleotides and reiterative cycles of binding, selection, and amplification to enrich for preferred ligand-binding sequences (Fig 1) [31–33]. Note that all these conventional combinatorial methods rely on the physical separation of ligand-bound from unbound nucleic acids, either through the use of altered physical properties (*e*.*g*., increased hydrophobicity, decreased electrophoretic mobility) or different affinity methods (*e*.*g*., biotin-avidin interactions, immunoprecipitation). Thus, conventional combinatorial selections require some foreknowledge of the ligand being employed in order to achieve effective separation of bound and unbound populations. Our laboratory has developed a unique combinatorial approach, Restriction Endonuclease Protection, Selection and Amplification (REPSA), that relies on a ligand-dependent inhibition of an enzymatic template inactivation process to achieve selection rather than by any physical separation means [34]. For REPSA this entails ligand-dependent inhibition of type IIS restriction endonucleases (IISRE), which cleave double-stranded DNA without sequence specificity at a fixed distance from their recognition sequence [35]. As REPSA does not require any foreknowledge of the ligands under investigation, it can easily work with mixed populations of different ligand types (*e*.*g*., proteins, nucleic acids, small molecules) under a variety of physiologically relevant conditions to provide useful information regarding each of their preferred binding sequences [34,36–41]. Thus REPSA is most amenable to discovery purposes, when little is known regarding the ligand(s) under investigation.

In the present report, we describe the application of REPSA to determine the preferred DNA-binding sequences for the *T*. *thermophilus* HB8 transcription regulatory protein encoded by the *TTHA0167* gene, SbtR. Over 10k selected DNAs were sequenced and a 14-mer sequence logo with extremely high significance *E* = 1.7 x 10^−2443^ was determined. Mapping these sequences to the *T*. *thermophilus* HB8 genome identified several promoter regions that should bind SbtR, these directing expression of genes encoding proteins with presumed transport, nucleic acid modification, and regulatory functions. This study provides a proof-of-concept that REPSA may be used to initiate our understanding of transcription regulatory proteins through the definition of their preferred DNA binding sequences, thereby leading to postulates of their potential biological functions.

## Results

### SbtR expression and characterization

SbtR (UniProtKB Q5SLX6) is the protein encoded by the *T*. *thermophilus* HB8 *TTHA0167* gene. It contains 189 amino acid residues and has an expected molecular mass of 21,539 Da. A Prosite scan of its protein sequence found a predicted TetR-type α-helix-turn-α-helix (HTH) motif from amino acids 13–73, with a DNA-binding H-T-H motif from amino acids 36–55. Such is consistent with a TetR/AcrR family transcriptional regulator protein [42]. Also noted in the SbtR sequence is a single cysteine residue at position 166. As TetR proteins normally exist as homodimers under physiological conditions, the location of C164 near the C-terminus suggests that it could participate in disulfide bond formation. A 2.05 Å crystal structure for SbtR presently exists (RCSB PDB 3VUQ) [24]. Its asymmetric unit is comprised of two SbtR homodimers. Each of these possesses the three dimensional structure characteristic of TetR family proteins, specifically the two alpha helices defining a HTH motif. Notably, this structure does provide evidence for an intermolecular disulfide bond between the C164 residues in each homodimer.

**Fig 2:**
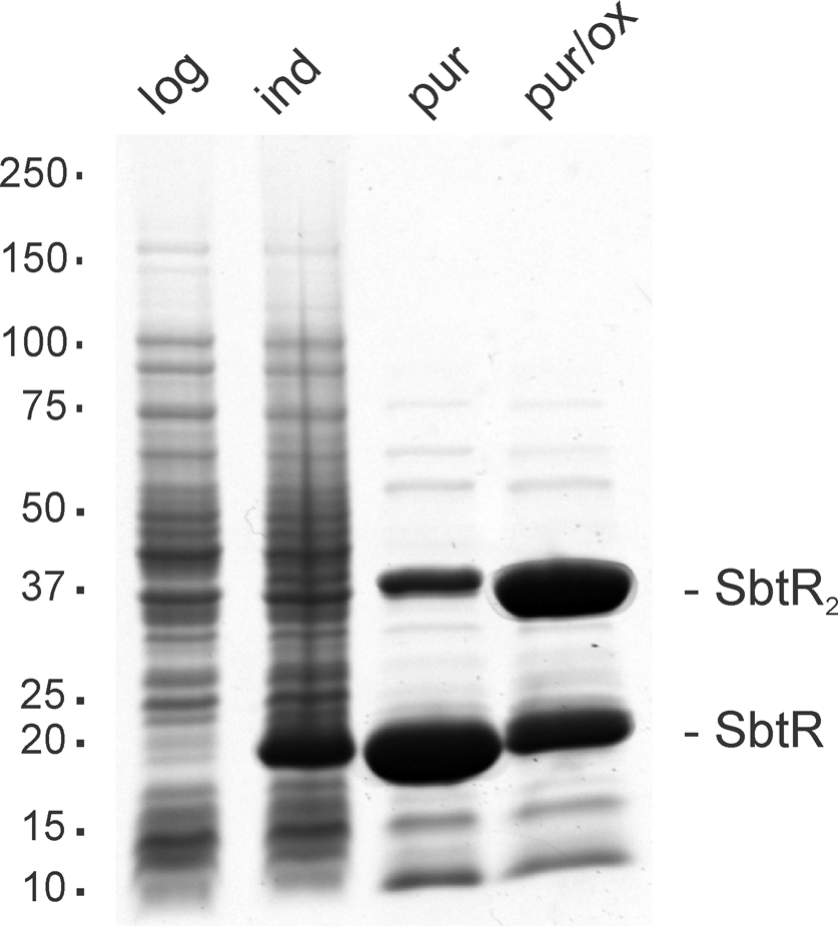
Expression and purification of SbtR protein. Shown is a Coomassie Brilliant Blue R250-stained 4–20% SDS-PAGE gradient gel onto which was loaded whole cell extracts or partially purified fractions containing SbtR protein. Lanes shown left to right: (log) 14 μg whole cell extract from logarithmic growth *E coli* BL21(DE3) bacteria containing the plasmid pET-sbtR, (ind) 24 μg whole cell extract from the aforementioned bacteria following IPTG-induction, (pur) 36 μg purified SbtR protein loaded under standard reducing conditions, (pur/ox) as previous, except that the sample was loaded under oxidizing conditions. The location of molecular weight standards is indicated at the left of the figure. SbtR and SbtR_2_ indicate the locations of reduced monomeric and oxidized dimeric SbtR proteins, respectively.

*E*. *coli* strain BL21(DE3), transformed with the plasmid pET-sbtR, was used to express SbtR protein. Following induction, whole cell extracts were prepared and then heat-treated to denature *E*. *coli* proteins. Given the thermostability of SbtR, it remained soluble, allowing the facile removal of most contaminating *E*. *coli* proteins by centrifugation. This purified SbtR preparation used in our studies was found to contain two dominant protein species of apparent molecular masses 19- and 38-kDa as indicated by SDS-PAGE (Fig 2, lane 3). These likely corresponded to the monomeric and disulfide-linked dimeric forms of SbtR, as evident by the increase in the latter species when Laemmli loading buffer lacking reductant was used in sample preparation (Fig 2, lane 4). Overall our stock SbtR was estimated to be greater than 90% pure with the majority being the disulfide-linked dimeric species.

### REPSA selection of SbtR-binding DNAs

**Fig 3:**
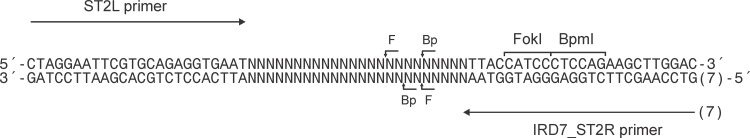
Sequence of the REPSA selection template ST2R24. Shown is the nucleotide sequence of the double stranded DNA used to initiate REPSA selections in this study. (N) Random nucleotides. The recognition sequences and cleavage sites of the type IIS restriction endonucleases FokI and BpmI are indicated by brackets and arrows, respectively. The sequences corresponding to the primers used to PCR amplify this selection template are indicated by horizontal arrows. Note that both the primer IRD700_ST2R and selection template ST2R24 were singly 5′-end labeled with the fluorophore IRDye 700 (7). This allowed their sensitive detection through native PAGE and infrared fluorescence imaging.

Selection template ST2R24 was designed for the selection of DNAs specifically recognized by prokaryotic DNA-binding proteins. A schematic of ST2R24 is shown in Fig 3. It was derived from the selection template ST2R14, which was successfully used to identify preferred binding sites for the human TATA-binding protein, and ST2R18, which was successfully used to identify preferred binding sites for the *E*. *coli* SlmA protein [36,41]. ST2R24 possesses the same flanking sequences as both ST2R14 and ST2R18, thereby allowing the use of IISREs FokI and BpmI for selection. FokI and BpmI were previously found to be the most effective IISREs for REPSA [36,38–41]. Additionally, the opportunity to use multiple IISREs was incorporated to provide maximal flexibility in REPSA selections and particularly to avoid selection of specific IISRE cleavage-resistant sequences [34]. Note that the longer (24 bp) randomized cassette was chosen to better permit the identification of prokaryotic DNA-binding proteins, given that most (89/93) *E*. *coli* transcription factors for which position-specific scoring matrices exist typically recognize binding sites of 24 bp or less [20]. One limitation of the longer randomized sequence is that there are more combinations possible in this sequence space (4^24^/2 = 1.4 × 10^14^) than would be present for the R14 or R18 templates (1.3 × 10^8^ and 3.4 × 10^10^, respectively). Thus, our starting population of ST2R24 DNA (42 fmoles or 2.5 × 10^10^ molecules) would not be expected to be a complete representation of all 24 bp sequences possible. However, although the average *E*. *coli* transcription factor binding site size may be 16.8 bp in length, only a subset of these bases typically is important in determining binding specificity [20]. Thus it was considered likely that useful sequence information could be obtained using the ST2R24 selection template in REPSA studies with prokaryotic transcription factors.

A single round of REPSA consists of three steps: ligand binding to the DNA pool, cleavage of unbound DNA by IISRE, and amplification of uncleaved DNA by PCR (Fig 1). For each REPSA round, three reactions were prepared and run in parallel: a DNA control containing SbtR but not subject to IISRE cleavage, a IISRE cleavage control lacking SbtR, and a selection reaction containing SbtR and subjected to IISRE cleavage. An aliquot from the SbtR/IISRE selection reaction would then be PCR amplified a total of 6, 9, and 12 cycles, to ensure that the maximal quantity of fully annealed duplex selection template product would result. Products from the binding/cleavage reactions and PCR reactions were analyzed after each REPSA round by native PAGE and IR fluorescence. If evidence for ligand-dependent, IISRE cleavage-resistant DNA was observed, further REPSA selections were discontinued, and DNA subjected to massively parallel sequencing. Otherwise, the PCR output of one round would then be used to seed a subsequent REPSA round: binding, cleavage, and amplification.

**Fig 4:**
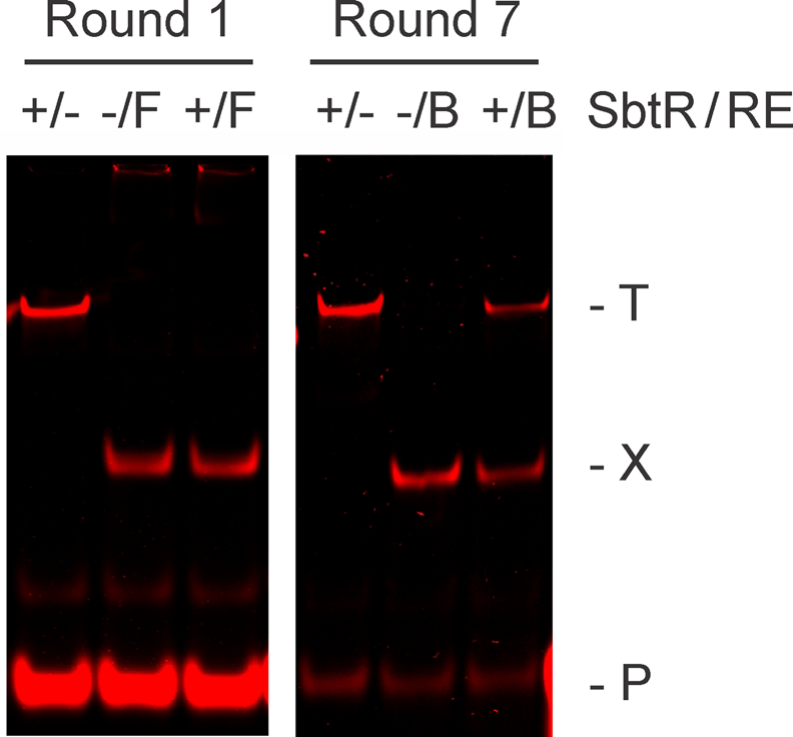
REPSA section of SbtR-dependent IISRE cleavage-resistant DNA species. Shown are LICOR Odyssey images of restriction endonuclease cleavage protection assays during Round 1 (left panel) and Round 7 (right panel) of REPSA selection with 40 nM SbtR protein. The presence of SbtR or IISRE FokI (F) or BpmI (B) is indicated above each lane. Lanes include: (+/-) total DNA control, (-/F or -/B) IISRE cleavage control, and (+/F or +/B) IISRE selection with SbtR. The electrophoretic mobility of the intact (T) and cleaved (X) selection template, as well as the IRD7_ST2R primer (P), are indicated at right of figure.

For our selections of SbtR-binding DNAs, a total of seven rounds of REPSA were performed before evidence of a SbtR-dependent, IISRE cleavage-resistant DNA population was observed. Such may be seen by comparing the DNA products of SbtR-binding/IISRE-cleavage reactions from Round 1 and Round 7 (Fig 4). Under these reaction conditions approximately half of the input DNA from Round 7 was not cleaved by the IISRE BpmI when 18 nM SbtR dimer was present. This level of protection is comparable with prior REPSA studies and has been found indicative of a majority of DNAs possessing preferred ligand-binding sites [34,38–40]. Note that levels of remaining IRD7_ST2R primer DNA differed in the reaction products obtained from Rounds 1 and 7. These were the result of limiting PCR amplifications used during REPSA and were not found to appreciably affect the selection process. Also note, Rounds 1–5 were performed using the IISRE FokI while Rounds 6 and 7 were performed with the IISRE BpmI. This was necessary given the unexpected appearance of SbtR-independent, FokI cleavage-resistant DNA following Round 5 selection (data not shown), which precluded continued use of this IISRE in subsequent selections.

**Fig 5:**
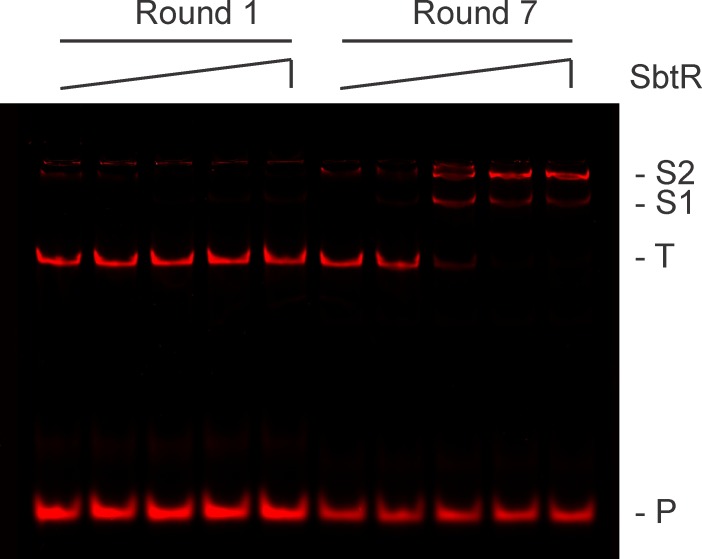
Validation of REPSA-selected SbtR-binding DNA species. Shown are LICOR images of electrophoretic mobility shift assays containing pooled DNA from either Round 1 (left lanes) or Round 7 (right lanes) of REPSA selection and different concentrations of SbtR protein (from left to right: 0, 1, 10, 100, or 1000 nM SbtR dimer). The electrophoretic mobility of two protein-DNA complexes (S2 and S1) as well as uncomplexed ST2R24 selection template (T) and IRD7_ST2R primer (P) are indicated at right of figure.

Before massively parallel sequencing REPSA selected DNAs, it was important to independently determine whether the selected DNAs contained *bona fide* high-affinity SbtR binding sequences. To best demonstrate this, we chose to analyze the selected DNA pool using an electrophoretic mobility shift assay (EMSA). 21 fmoles of PCR DNA product from either Round 1 or Round 7 was incubated increasing concentration of SbtR protein under conditions to permit specific DNA binding. DNA species, including SbtR-DNA complexes, were resolved by native 10% PAGE and visualized by IR fluorescence. As shown in Fig 5, no evidence for SbtR-DNA complexes was observed with Round 1 DNA, even at high (1000 nM) SbtR dimer concentrations. This indicates that SbtR does not form electrophoretically stable complexes with nonspecific DNAs under our reaction conditions. However, with Round 7 DNA, most was present in one of two different SbtR-DNA complexes, even at concentrations as low as 10 nM SbtR dimer. Such was considered good evidence that the majority of the Round 7 DNAs contained stable, high-affinity SbtR-binding sites and was worthy of massively parallel sequencing.

### Sequencing and analysis of REPSA-selected, SbtR-binding DNAs

In this study we obtained sequence information from REPSA selected DNAs using massively parallel sequencing. Such is far more efficient than the subcloning and individual sequencing employed previously and allows the analysis of thousands of sequences rather than dozens [43]. To begin this process, DNA from Round 7 REPSA selection were used to generate an amplicon library suitable for sequencing on the Ion Personal Genome Machine (Ion PGM). Amplicon libraries were prepared using fusion PCR primers, which contained the conserved flanking regions of our ST2R24 selection template appended to sequences directing annealing to either Ion individual sequencing particles (ISPs) or sequencing primers. After synthesis, the fusion PCR products were quantified by Qubit dsDNA HS fluorescence assay and analyzed by native PAGE/ethidium bromide staining to determine their quality. Note that the fusion PCR primer that contains the sequencing template (A_BC04_ST2R) also contained an Ion Xpress barcode sequence. Such allowed multiplexing of experiments during ISP preparation and sequencing when different barcodes were used.

Massively parallel sequencing using the Ion PGM system is a multistep process involving library preparation, template-positive ISP preparation and purification, semiconductor-based sequencing using the Ion PGM, and sequence analysis using the Ion Torrent server [44]. After fusion library preparation, all subsequent steps followed standard operating procedures as described in the Materials and Methods. Quality control assessments were made upon the synthesized fusion library and template-positive ISPs before sequencing. Quality assessments on the sequencing runs themselves were provided in real time by a web portal to the Ion Torrent server. Routinely we obtain 220k – 540k reads of ~50 bp mean length, having 6.8M to 19.1M bases with ≥Q20 quality scores, far more than is necessary for their subsequent analysis. Thus most all of our sequencing runs were constituted from 4~5 individual experiments, using barcoded libraries to permit their discrimination. For the results described in this paper, SbtR-selected DNA were one of four experiments sequenced in parallel. For the SbtR DNA alone, Ion PGM sequencing yielded 3,215,262 bases, 2,376,479 ≥ *Q*20, resulting in 62,891 reads of 51 bp mean read length.

The Ion Torrent Suite of software packages and affiliated Ion Reporter software provide a wealth of analysis options on the output from the Ion PGM. Unfortunately, packages were not available for converting Ion PGM sequencing data into a format suitable for identifying common sequence patterns (motifs) using software such as MEME Suite [45]. To make this possible, a simple script was written that takes a fastq file of Ion PGM sequencing data, identifies those sequences that contain both intact ST2R and ST2L complement flanking sequences and an appropriate number (24) of intervening bases, and collects them in a file formatted for input into MEME Suite analysis software. These scripts (Sequencing1.java and parameter.txt) are freely available upon request. Typically we found that 33k – 63k Ion PGM reads yielded 3.1k – 7.4k (~5–10%) useful sequences following this step. Specifically for SbtR-selected DNAs, 62,891 Ion PGM reads yielded 3,059 sequences after processing. Of these, two were found in triplicate and 64 in duplicate, giving 2,991 unique sequences. The reason for this large attrition may be due in part to incomplete sequencing products and sequencing misreads either within the flanking sequences or randomized cassette region. Massively parallel sequencing has a far higher error rate than conventional Sanger sequencing and the semiconductor sequencing used by the Ion PGM has a tendency to inaccurately call multiple, contiguous bases [46]. Nonetheless, a single Ion 314 chip would yield more than sufficient data such that ten REPSA experiments could be multiplexed at the same time.

**Fig 6:**
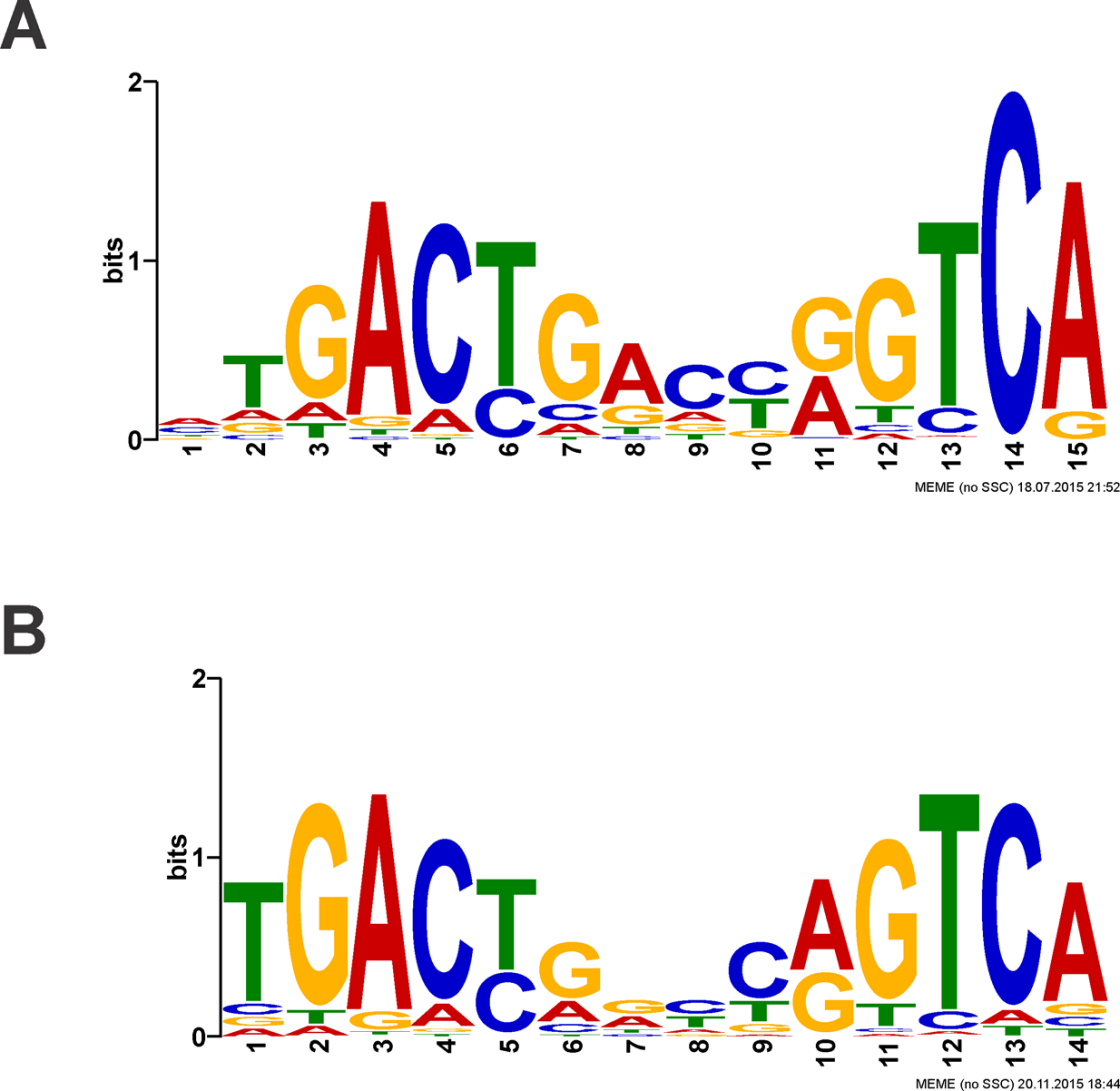
Sequence logos of REPSA-selected SbtR-binding sequences. Sequence logos were determined using MEME software with inputs of 1000 Round 7 DNA sequences. (**A**) MEME performed with no filters. (**B**) Palindromic filter.

The MEME Suite is a series of motif-based sequence analysis tools that allows one to discover motifs in unaligned DNA sequences as well as to identify locations of these motifs in a sequence database or genome [45]. We used the web version 4.10.2 of Multiple Em for Motif Elucidation (MEME) to discover protein binding motifs in our REPSA selected DNA sequences. Input was the first 1000 sequences obtained from our Sequencing1.java output file, the maximum accommodated by MEME. All defaults were used (*e*.*g*., normal mode of motif discovery, site distribution of zero or one occurrence per sequence, identify three best motifs, 0-order model of sequences as background) with the exception that the search be restricted to palindromes where indicated. For our Round 7 REPSA selected SbtR-binding sequences, a nonpalindromic MEME analysis identified a single 15-mer motif that was present in the vast majority of the sequences (929/1000). The statistical significance of this motif was extraordinarily high, as measured by its E-value (8.7e-2485), the expected number of motifs with the given log likelihood ratio or higher, and with the same width and site count, which one might find in a similarly sized set of random sequences. A sequence logo of its position weighted matrix is shown in Fig 6A. As most TetR-family HTH proteins exist as homodimers and recognize palindromic binding sites, we repeated the MEME analysis with a limit to palindromic sequences advanced option. This analysis found a 14-mer motif (Fig 6B) in 938/1000 sequences with an E-value of 1.7e-2443, still extraordinarily significant. From this motif a 14-bp SbtR-binding consensus sequence could be derived, 5′-TGACTGGCCAGTCA-3′. Note that additional MEME analyses were performed with subsequent sets of 1000 sequences from our Sequencing 1.java output file. In all cases very similar results were obtained (data not shown). Taken together, these analyses strongly suggest that the identified sequences correspond to high-affinity SbtR binding sequences. Further, it may be possible to identify those individual bases that are most important in SbtR-DNA recognition as those most prominently represented in these sequence logos.

### Biophysical validation of SbtR-binding DNAs

With a SbtR sequence logo in hand, it was necessary to determine its validity. Three different binding assays were employed: IISRE cleavage-protection (REPA), EMSA, and biolayer interferometry (BLI). REPA is directly comparable to the selections performed in REPSA and would provide an indication how well SbtR bound to its consensus sequence inhibits a challenge by a IISRE. EMSA is a well-established electrophoretic assay that should provide information on the quantity and quality of stable SbtR-DNA complexes. Finally, BLI is a biophysical method that has more recently been applied to the study of protein-DNA complexes [47]. It was used as a cost-effective means of determining kinetic parameters (*k*_on_, *k*_off_) and equilibrium binding constants of SbtR binding to a consensus sequence as well as various point mutants. Note that the latter study will allow us to directly test the hypothesis that those bases most represented in the sequence logo correspond to those most important in the determining the binding affinity to this sequence.

**Fig 7:**
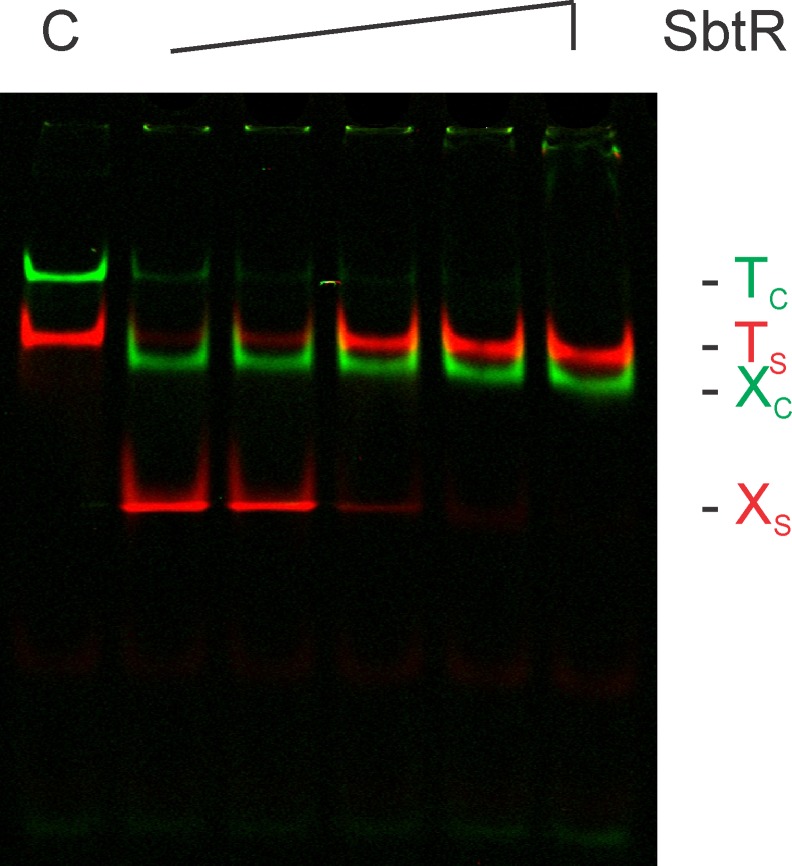
SbtR-binding to a consensus sequence as analyzed by a restriction endonuclease protection assay. Shown are LICOR images of IRD7-labeled SbtR consensus DNA (red) and IRD8-labeled REPSAis control DNA (green) subjected to BpmI cleavage following binding reactions in the presence of (left to right) 0, 1, 10, 100, 1000 nM dimer SbtR protein. (C) Uncleaved DNA control lane. (T) Intact, uncleaved DNA, (X) cleaved DNA.

In order to perform REPA, a suitable DNA probe was constructed containing the 14-mer SbtR consensus, 5′-TGACTGGCCAGTCA-3′, flanked by ST2L and IRD7_ST2R sequences. This allowed facile synthesis of a 63-bp IRD700 fluorophore-labeled probe by PCR, SbtR consensus. A second 86-bp IRD800 fluorophore-labeled probe containing an unrelated 14-bp sequence was synthesized using the oligonucleotides REPSAis, ST2R, and IR8_trP1_ST2L (Table 1). This DNA, REPSAis control, served as an internal IISRE cleavage control in each REPA assay. Reactions containing 1 nM each SbtR consensus and control DNAs were incubated with increasing concentration of SbtR under suitable binding conditions, then subjected to cleavage by the IISRE BpmI. As shown in Fig 7, over 90% of the control DNA (green) was cleaved whenever BpmI was present, indicating that adequate IISRE was present to cleave DNA with single-hit kinetics under our reaction conditions. Interestingly, increased levels of control DNA cleavage was noted upon increasing SbtR concentrations, up to 1000 nM dimer final. Such was contrary to the supposition that SbtR has sufficient binding affinity to nonspecific DNA such that it could inhibit cleavage on these DNAs. Rather, it is suggestive that the presence of additional protein increased overall IISRE cleavage efficiency, potentially by stabilizing BpmI protein or rendering more of the relatively hydrophobic IRD800 fluorophore-labeled DNA accessible to cleavage. In the absence of SbtR, consensus DNA (red) was also ≥90% cleaved under our reaction conditions. However, in the presence of 10 nM dimer SbtR, ≥90% of the consensus DNA was protected from cleavage. Such indicates that the equilibrium binding constant for SbtR to its consensus sequence is between 1 nM and 10 nM dimer under our reaction conditions. Note that while a REPA assay employing a finer titration of SbtR concentrations could conceivably be used to better ascertain its equilibrium binding constant to consensus DNA, such was not performed. The two-step REPA assay (ligand binding, IISRE cleavage) is more complicated that more direct binding assays used (EMSA, BLI) and has the additional complication that IISREs need not be completely innocuous probes of ligand binding, as their intrinsic DNA affinity can compete for DNA binding.

**Table 1:** Oligonucleotides.

Name	Sequence	Length	Purif.	Use
ST2R24	CTAGGAATTCGTGCAGAGGTGAATNNNNNNNNNNNNNNNNNNNNNNNNTTACCATCCCTCCAGAAGCTTGGAC	73	PAGE	REPSA selection template precursor
ST2L	CTAGGAATTCGTGCAGAGGTGAAT	24	Desalt	PCR primer
ST2R	GTCCAAGCTTCTGGAGGGATGGTAA	25	Desalt	PCR primer
IRD7_ST2R	/5IRD700/GTCCAAGCTTCTGGAGGGATGGTAA	25	HPLC	5′ IRDye 700-modified PCR primer
A_BC01_ST2R	CCATCTCATCCCTGCGTGTCTCCGACTCAGCTAAGGTAACGATGTCCAAGCTTCTGGAGGGATG	64	PAGE	Fusion PCR primer
trP1_ST2L	CCTCTCTATGGGCAGTCGGTGATCTAGGAATTCGTGCAGAGGTGA	45	PAGE	Fusion PCR primer
A_uni	CCATCTCATCCCTGCGTG	18	Desalt	PCR primer
trP1_uni	CCTCTCTATGGGCAGTCGG	19	Desalt	PCR primer
IRD8_trP1_ST2L	/5IRD800/CCTCTCTATGGGCAGTCGGTGATCTAG	27	HPLC	5′ IRDye 800-modified PCR primer
REPSAis	CTAGGAATTCGTGCAGAGGTGAATCGTCATAGAATTCGTTACCATCCCTCCAGAAGCTTGGAC	63	PAGE	REPSAis control DNA precursor
Bio_ST2R	/5BiodT/GTCCAAGCTTCTGGAGGGATG	22	HPLC	5′ biotin-modified PCR primer
ST2_SbtR_R7_wt	AGGAATTCGTGCAGAGGTGAATTGACTGGCCAGTCATTACCATCCCTCCAGAAGCTTGG	59	Desalt	SbtR consensus DNA probe precursor
ST2_SbtR_R7_m1	AGGAATTCGTGCAGAGGTGAATAGACTGGCCAGTCATTACCATCCCTCCAGAAGCTTGG	59	Desalt	SbtR mutant 1 DNA probe precursor
ST2_SbtR_R7_m2	AGGAATTCGTGCAGAGGTGAATTCACTGGCCAGTCATTACCATCCCTCCAGAAGCTTGG	59	Desalt	SbtR mutant 2 DNA probe precursor
ST2_SbtR_R7_m3	AGGAATTCGTGCAGAGGTGAATTGTCTGGCCAGTCATTACCATCCCTCCAGAAGCTTGG	59	Desalt	SbtR mutant 3 DNA probe precursor
ST2_SbtR_R7_m4	AGGAATTCGTGCAGAGGTGAATTGAGTGGCCAGTCATTACCATCCCTCCAGAAGCTTGG	59	Desalt	SbtR mutant 4 DNA probe precursor
ST2_SbtR_R7_m5	AGGAATTCGTGCAGAGGTGAATTGACAGGCCAGTCATTACCATCCCTCCAGAAGCTTGG	59	Desalt	SbtR mutant 5 DNA probe precursor
ST2_SbtR_R7_m6	AGGAATTCGTGCAGAGGTGAATTGACTCGCCAGTCATTACCATCCCTCCAGAAGCTTGG	59	Desalt	SbtR mutant 6 DNA probe precursor
ST2_SbtR_R7_m7	AGGAATTCGTGCAGAGGTGAATTGACTGCCCAGTCATTACCATCCCTCCAGAAGCTTGG	59	Desalt	SbtR mutant 7 DNA probe precursor
IRD7_ST2_SbtR_mini_2a	/5IRD700/GCCCTGACTGGCCAGTCACCCG	22	HPLC	SbtR mini probe
ST2_SbtR_mini_2b	CGGGTGACTGGCCAGTCAGGGC	22	Desalt	SbtR mini probe

(N) Random nucleotides. Length is in nucleotides.

**Fig 8:**
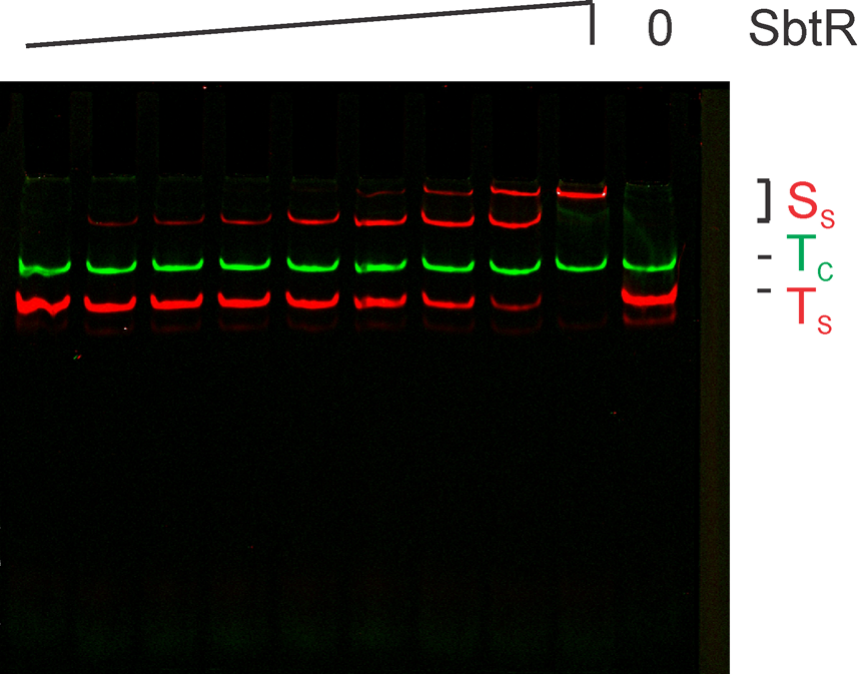
SbtR-binding to a consensus sequence as analyzed by an electrophoretic mobility shift assay. Shown are LICOR images of IRD700-labeled SbtR consensus DNA (red) and IRD800-labeled REPSAis control DNA (green) incubated with (left to right) 0.0625, 0.125, 0.25, 0.5, 1, 2, 4, 8, 16 nM dimer SbtR protein. (0) uncomplexed DNA control lane. (S) SbtR-DNA complexes, (T) uncomplexed DNA.

For our EMSA assays, the same DNA probes used in our REPA assays, 63-bp IRD700-labeled SbtR consensus and 86-bp IRD800-labeled REPSAis control, as well as the identical binding conditions, were used. Afterward, the different DNA species were resolved by native PAGE and visualized by IR fluorescence. Based on our REPA experiments, two-fold serial dilutions of SbtR dimer from 0.0625 nM to 16 nM were incubated with 1 nM each consensus (red) and control (green) DNAs to permit specific binding. As shown in Fig 8, no diminution of control DNA fluorescence was observed at its expected mobility. Likewise, no new species were observed. Taken together, these data would indicate that under our reaction conditions no electrophoretically stable SbtR-control DNA complex exists. However, such was not the case with consensus DNA, which demonstrated the appearance of a new, slower mobility species (S_1_) at concentrations as low as 0.25 nM SbtR dimer and an even slower mobility species (S_2_) at 4.0 nM SbtR dimer. Using a simple binding equilibrium and quantitation of our EMSA data, one can approximate the K_D_ for the S_1_ SbtR-consensus DNA complex as 2.8 nM. Similarly, one may approximate the K_D_ for the S_2_ complex as ~8 nM.

**Fig 9:**
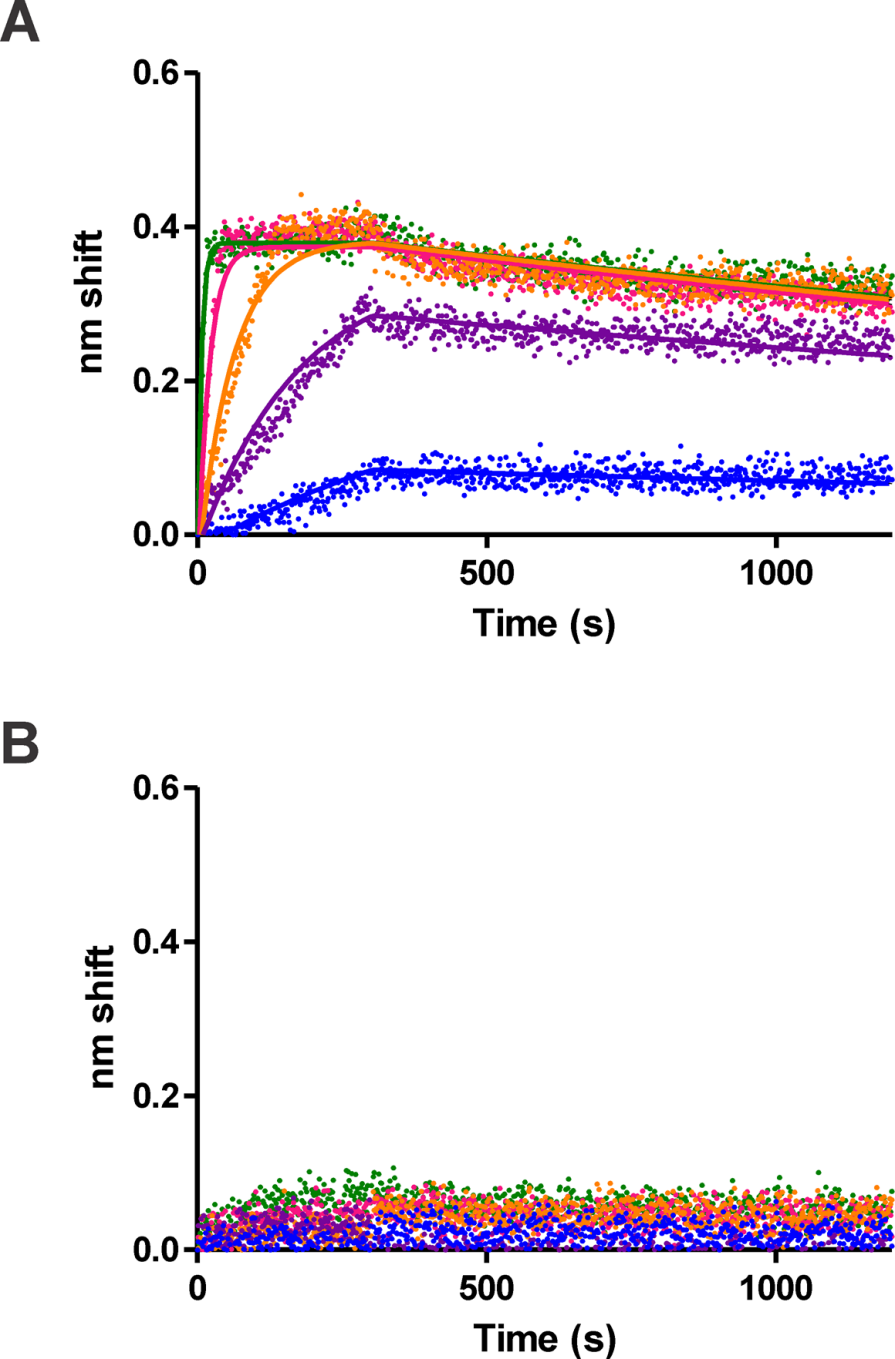
SbtR-binding to a consensus sequence as analyzed by biolayer interferometry. Shown are raw traces (dots) and best-fit lines generated by GraphPad Prism using data obtained from a fortéBIO Octet biolayer interferometer. Binding reactions contained (top to bottom) 150, 50, 16.7, 5.56, 1.85 nM SbtR dimer protein. (**A**) Target was biotinylated ST2 DNA containing the 14-bp consensus insert 5′-TGACTGGCCAGTCA-3′. (**B**) Biotinylated ST2 control DNA.

While the values derived from our EMSA experiments are useful measures of SbtR-DNA binding, greater accuracy and precision necessitate the use of alternative assays, especially those that do not require maintenance of complex stability during gel electrophoresis. To accomplish this, we used biolayer interferometry (BLI), which measures changes in an optical interferometric profile upon macromolecular binding to a biosensor [48]. Biotinylated 63-bp DNAs corresponding to SbtR consensus and REPSAis control were synthesized by PCR and used as probes for BLI. These were allowed to bind to streptavidin–coated biosensors before the addition of different concentrations of SbtR. Both association and subsequent dissociation were monitored in real time. Data from these time points were analyzed using single-state association then dissociation kinetics. Individual values were first calculated for each SbtR concentration investigated and average values determined for those concentrations that provided unambiguous results (typically SbtR concentrations > 2 nM dimer). As shown in Fig 9A, SbtR demonstrated a concentration-dependent rate association to its consensus sequence and uniformly slow dissociation rate. These were accurately modeled using single-state association and dissociation kinetics, with an average goodness-of-fit determination R^2^ = 0.924. Association and dissociation rates could be derived from these models as well as equilibrium binding constants calculated. These values are provided in Table 2. Comparable experiments with REPSAis control DNA were performed; however, they showed no evidence for SbtR binding through the concentration range investigated (Fig 9B). Taken together, our data show that SbtR bound its REPSA-identified consensus sequence with high affinity and specificity, much as would be expected for a HTH-motif transcriptional regulator.

**Table 2:** SbtR-DNA Binding Parameters.

Name	Sequence	*k*_on_ (M^-1^s^-1^)	*k*_off_ (s^-1^)	K_D_ (M)	R^2^
wt	TGACTGGCCAGTCA	990067	0.0002144	2.166e-10	0.9239
m1	aGACTGGCCAGTCA	488986	0.0002692	5.506e-10	0.9793
m2	TcACTGGCCAGTCA	288966	0.002055	7.111e-9	0.9838
m3	TGtCTGGCCAGTCA	423852	0.0009222	2.176e-9	0.9415
m4	TGAgTGGCCAGTCA	427439	0.0005304	1.241e-9	0.9766
m5	TGACaGGCCAGTCA	265994	0.004134	1.554e-8	0.9709
m6	TGACTcGCCAGTCA	1097e+6	0.0001747	1.593e-10	0.8912
m7	TGACTGcCCAGTCA	577799	0.0004846	8.387e-10	0.9526

Lowercase nucleotides indicates mutation from consensus SbtR sequence.

In our hands, BLI provided the best quantitative data for investigating SbtR-DNA binding. Thus we used BLI to investigate mutations of the SbtR consensus sequence, to determine their importance in determining binding affinity and specificity. Oligonucleotides containing single point mutations in the SbtR consensus sequence were obtained and biotinylated probes generated by PCR as described. A list of the oligonucleotide sequences used is provided in Table 1. Point mutations were chosen as the complement of the most highly represented base at each position within one-half of the SbtR palindromic sequence logo, with the belief that they would be the most disruptive. BLI experiments were performed as described previously and the derived binding parameters shown in Table 2. We found that association rates varied over a four-fold range, from 265994 M^–1^s^–1^ (m5) to 1097000 M^–1^s^–1^ (m6), while dissociation rates varied over a far larger (24-fold) range, from 0.0001747 s^–1^ (m6) to 0.004134 s^–1^ (m5). Equilibrium binding constants ranged from 1.554× 10^−8^ M (m5) to 1.593 × 10^−10^ M (m6), the former being 72-fold worse than the consensus sequence (K_D_ = 2.166 × 10^−10^ M), while the latter being slightly better. In general, mutations of bases with the greatest significance in the sequence logo (*e*.*g*., positions 1–5) were found to have the greatest effect on binding affinity. However, mutations in positions 5 and 7 had greater effects than would have been anticipated from the sequence logo data, while position 1 had a lesser effect. This may reflect the choice of mutation rather than the position itself, given that the mutant base still has its original base on the opposite strand. For some positions, this could be sufficient to provide some binding affinity/specificity.

**Fig 10:**
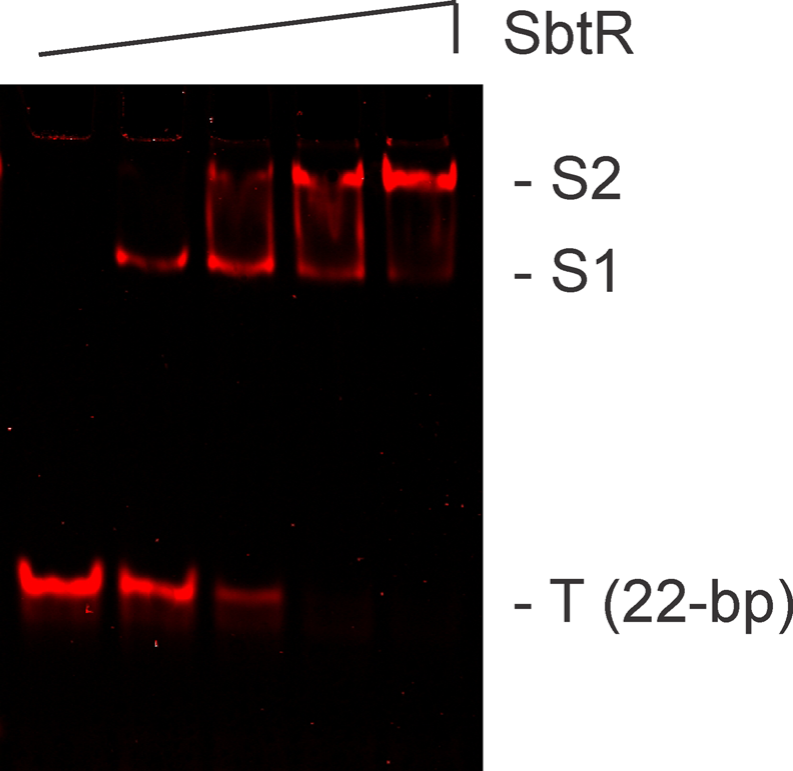
SbtR-binding to a minimal consensus sequence as analyzed by an electrophoretic mobility shift assay. Shown are LICOR images of IRD700-labeled SbtR consensus DNA (red) incubated with (left to right) 2, 20, 200, 2000 nM dimer SbtR protein. (0) uncomplexed DNA control lane. (S) SbtR-DNA complexes, (T) uncomplexed DNA.

The appearance of two SbtR-DNA complexes was unexpected and curious. One hypothesis is that the relatively large flanking regions in our DNA probes facilitated binding of a second SbtR dimer at high concentrations. While our REPSAis control DNA, which contains the identical flanking sequences as our SbtR consensus DNA, did not show any evidence for cryptic SbtR binding sites, we undertook an investigation using a shorter DNA probe. A 22-bp DNA probe containing the 14-bp SbtR consensus sequence and 4-bp G/C-rich flanking sequences necessary to maintain stability at 55°C was assembled using the oligonucleotides IRD7_ST2_SbtR_mini_2a and ST2_SbtR_mini_2b. In an EMSA experiment (Fig 10), SbtR was able to bind the SbtR mini probe with an apparent K_D_ of 5 nM for the faster migrating S1 complex. More important, the shorter SbtR mini probe did form the slower migrating S2 complex, with an apparent K_D_ of 30 nM. Note that both of these values are comparable to those found with the 63-bp SbtR consensus probe. These data suggest that the longer flanking sequences are not the primary determinant in determining the affinity of SbtR for its consensus sequence and for the formation of the S2 complex. Further studies will be necessary to determine the exact nature of this species.

### Bioinformatics of SbtR-binding to the *T*. *thermophilus* genome

Having determined a SbtR consensus sequence, it was possible to use the MEME Suite program Find Individual Motif Occurrences (FIMO) to identify matching sites within the *T*. *thermophilus* HB8 genome [45]. The position-specific frequency matrix corresponding to our best possible sequence match 5′-TGACTGGCCAGTCA-3′ was directly imported into FIMO from our palindromic MEME search and used to probe the GenBank *Thermus thermophilus* HB8 uid13202 version 210 database using default parameters. Output was 114 motif occurrences with a threshold probability of a random sequence of the same length matching the identified position with as good or better score (*P*-value) being less than 0.00001. The top 40 were subjected to further evaluation. Table 3 shows a list of these, removing duplicates that map for the same gene. For this subset, *P*-values ranged from 3.9 × 10^−8^ to 3.22 × 10^−5^, while *q*-values, a measure of false discovery rate, ranged from 0.0825 to 1. These sequences were then mapped by hand to their corresponding sites within the *T*. *thermophilus* HB8 genome (KEGG T00220, ttj), to identify genes/operons that could potentially be regulated by SbtR.

**Table 3:** FIMO of Best Possible Match TGACTGGCCAGTCA.

Start	End	p-value	q-value	Sequence	Location	Pro?	Gene
1254811	1254824	3.9e-08	0.0825	TGACCGGTCAGTCA	–17	Y	*TTHA1315*
				TGACTGACCGGTCA	–38	Y	*TTHA1316*
1284539	1284552	2.25e-07	0.238	TGACCGGTCGGTCA	+2	N	*(TTHA1342)*
1270098	1270111	2e-06	1	TGACCCGTTGGTCA	–90	~	*TTHA1330*
1705170	1705183	2.87e-06	1	TGACCCTTTGGTCA	–4	Y	*TTHA1821*
				TGACCAAAGGGTCA	–14	Y	*TTHA1822*
547821	547834	3.42e-06	1	TGACCGGCGGGTCA	+330	N	*TTHA0579*
1736492	1736505	3.42e-06	1	TGACCGGCGGGTCA	–15	N	*(TTHA1852)*
28295	28308	3.96e-06	1	TGACCCGCTGGTCA	–42	Y	*(TTHA0027)*
1526718	1526731	7.3e-06	1	TGACCGGTCAGTAT	+29	Y	*TTHA1605*
				ATACTGACCGGTCA	–45	Y	*TTHA1606*
700149	700162	8.79e-06	1	TGACTAAATAGTTG	–24	Y	*TTHA0733*
				CAACTATTTAGTCA	–123	~	*TTHA0734*
1264565	1264578	1.13e-05	1	TGACCTCTTGGTCA	+177	N	*TTHA1325*
10108	10121	1.26e-05	1	TGACTTTAGGGTCA	+868	N	*TTHAr01*
351149	351162	1.26e-05	1	TGACCCTAAAGTCA	+868	N	*TTHAr05*
669059	669072	1.26e-05	1	TGACCAGTTGCTCA	–21	Y	*(TTHA0706)*
754466	754479	1.84e-05	1	TGACCCGCGGGTCA	–21	Y	*(TTHA0787)*
1696587	1696600	1.84e-05	1	gaACTGGTCGGTCA	+1019	N	*TTHA1813*
1408945	1408958	1.93e-05	1	TGACCAGCTGGTCC	+195	N	*(TTHA1480)*
470721	470734	2.06e-05	1	TGACCTTTTGGTaA	+1413	N	*TTHA0506*
519878	519891	2.95e-05	1	TGACgATCTGGTCA	+534	N	*TTHA0558*
911715	911728	3.13e-05	1	CtACTGGATGGTCA	+108	N	*(TTHA0967)*
135727	135740	3.22e-05	1	TtACgAGCCAGTCA	–808	N	*TTHB146*

(*P*-value) Defined as the probability of a random sequence of the same length matching that position of the sequence with as good or better score. (*Q*-value) False discovery rate if the occurrence is accepted as significant. (Sequence) Lowercase indicates low-affinity binding site mutations. (Pro?) Promoter identified proximally upstream of the gene. (Gene) Parentheses indicate a gene located in the subsequent portion of a postulated operon. (~) Indicates that while a promoter is present, the SbtR-binding site does not overlap.

Multiple levels of consideration were used in evaluating candidate SbtR-regulated genes: (1) expected affinity of SbtR for the genomic sequence, (2) presence of a SbtR binding site within an identifiable promoter, and (3) SbtR regulating either an independent transcriptional unit or the first gene in an operon. With regards to expected SbtR binding affinity, genomic sites were compared to our sequence logo and BLI equilibrium binding affinity data, with those expected to have an affinity 10-fold lower than our consensus being flagged (Table 3, see sequences with lowercase). As FIMO ranks genomic sequences based on their match to the position-specific frequency matrix used to generate the sequence logo, and this is a fairly good representation of importance for each position in determining SbtR binding affinity, it is reasonable that most of the higher ranked sequences were not flagged. Such was more of concern for sequences whose *P*-values were greater than 1.5 × 10^−5^. Sequences ±200 bp of the genomic SbtR site was analyzed using both Softberry BPROM and University of Groningen PePPER to identify potential promoters [49,50]. Those with high scoring promoters are indicated in Table 3. Notably, 11 of the top 18 sequences were present in identifiable promoters. Finally, it was important to determine whether the potential SbtR-regulated gene was an individual transcriptional unit or part of an operon. Sequences ±20 kbp of the genomic SbtR site was analyzed using databases at National Autonomous University of Mexico (ProOpDB) and the University of Georgia (DOOR^2^) to identify possible operons [51,52]. Genes potentially regulated by SbtR that were initial members of operons or autonomous were well represented in the top sequence matches (13/18). However, it should be noted that being a downstream member of an operon does not preclude one from being independently transcriptionally regulated under certain circumstances, so long as a promoter is present.

**Fig 11:**
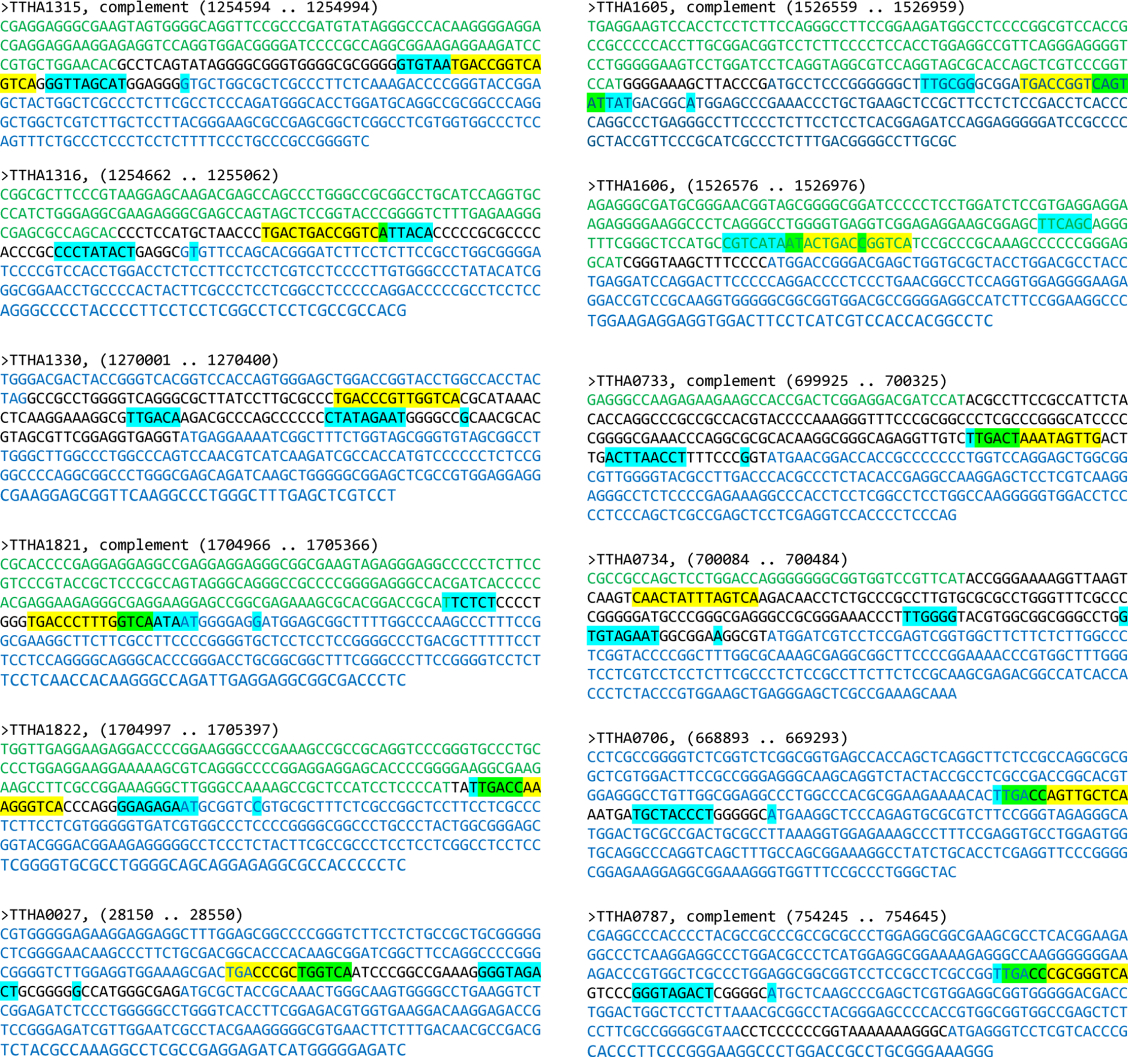
Bioinformatic identification of *T*. *thermophilus* HB8 promoters potentially regulated by SbtR. Shown are sequences +/- 200 bp of the first codon of a target gene identified through FIMO analysis as being potentially regulated by SbtR (see Table 2). Longest open reading frames with identical orientation as the target gene are indicated with blue nucleotides. Open reading frames with opposite orientation are indicated with green nucleotides. Black nucleotides indicate intragenic regions. Potential promoter elements (-30 and -10 boxes, +1 start site of transcription) were identified using Softberry BPROM and are indicated with blue highlighting. SbtR-binding sites are indicated with yellow highlighting. Regions of overlap between SbtR-binding sites and promoter elements are indicated by green highlighting.

Taking the above analyses into consideration, a set of 12 potential SbtR-regulated genes was identified. Fig 11 shows sequences ±200 bp from their start codons, identifying upstream genes with opposite orientation (green letters), genes with the same orientation (blue letters), SbtR matching sequence (yellow highlighting), and core promoter elements –35 box, –10 box and +1 sites (blue highlighting). In circumstances where core promoter elements and SbtR binding sites overlap, these were indicated with green highlighting. For our potential SbtR-regulated genes, 10 of 12 demonstrated SbtR binding sites that were overlapping and/or within their identified core promoter regions. These findings strongly suggest that SbtR may transcriptionally regulate these genes. Note: our analyses for core promoter elements may not identify all possible promoters. Additionally, a nearby SbtR binding site might interfere with other, unidentified transcriptional regulators. Thus, those two promoters that did not demonstrate an obvious role for SbtR regulation (genes *TTHA1330* and *TTHA0734*) may still be regulated in some fashion by this protein.

In a compact, prokaryotic genome, sets of contiguously aligned genes may be part of an operon and transcriptionally regulated simultaneously by a single promoter. Thus, to obtain a better understanding of the possible gene regulation by SbtR, analyses of operon structure in the vicinity of the 12 genomic SbtR binding sites described in Fig 11 was undertaken. Listed in Table 4 are the genes with SbtR binding sites identified within their promoters, the position of these genes within described transcriptional units and/or operons, and their protein names/postulated functions, as indicated by the KEGG and UniProtKB databases [53,54]. Note that two of these operons, starting with *TTHA1330* and *TTHA0734*, were not found to have their SbtR sites overlapping with identified core promoters. Thus, they are of lower confidence for being regulated by SbtR. Likewise, several genes that had overlapping SbtR/core promoter sequences (*e*.*g*., *TTHA0027*, *TTHA0706*, and *TTHA0787*), were found to be downstream members of identified operons. For these, it is unclear whether SbtR is an important transcriptional regulator for controlling their expression and what effect, if any, SbtR has on the expression of additional downstream genes.

**Table 4:** Potential SbtR-regulated Genes.

Promoter	Operon	Gene	Role
Y	N	*TTHA1315*	Putative integral membrane efflux protein
Y	N	*TTHA1316*	Uncharacterized protein (transmembrane, metalloendopeptidase)
~	1	*TTHA1330*	Branched-chain amino acid ABC transporter, periplasmic amino acid-binding protein
	2	*TTHA1331*	Branched-chain amino acid ABC transporter, permease protein
	3	*TTHA1332*	Branched-chain amino acid ABC transporter, permease protein
	4	*TTHA1333*	Uncharacterized protein
	*5*	*TTHA1334*	Branched-chain amino acid ABC transporter, ATP-binding protein
	*6*	*TTHA1335*	Branched-chain amino acid ABC transporter, ATP-binding protein
	*7*	*TTHA1336*	Peptide ABC transporter oligopeptide-binding protein
	*8*	*TTHA1337*	Peptide ABC transporter, permease protein
	*9*	*TTHA1338*	ABC transporter permease protein
	*10*	*TTHA1339*	Uncharacterized protein (secreted)
Y	1	*TTHA1821*	Uncharacterized protein (rRNA/tRNA-modification)
	2	*TTHA1820*	CinA-like protein (nicotinamide mononucleotide deamidase, ADP-ribose pyrophosphatase, pyridine nucleotide recycling)
	3	*TTHA1819*	RNA 2',3'-cyclic phosphodiesterase (2'-5'-RNA ligase)
	4	*TTHA1818*	Protein RecA (ATP-dependent DNA recombination/repair)
	5	*TTHA1817*	Ribonuclease Y (endoribonuclease, mRNA decay)
	6	*TTHA1816*	Rod shape-determining protein MreB (cell morphogenesis)
	7	*TTHA1815*	(3R)-hydroxyacyl-[acyl-carrier-protein] dehydratase FabZ (unsaturated fatty acid biosynthesis, lipid A biosynthesis)
	8	*TTHA1814*	Uncharacterized protein (RNA binding)
Y	1	*TTHA1822*	Probable transporter (transmembrane)
	2	*TTHA1823*	Putative hydrolase (phosphatase)
Y	(3)	*TTHA0027*	Probable potassium channel, beta subunit (oxidoreductase)
	*4*	*TTHA0028*	Putative macrolide-efflux protein (transmembrane)
Y	N	*TTHA1605*	Probable acylamino-acid-releasing enzyme (serine-type peptidase)
Y	1	*TTHA1606*	GTP cyclohydrolase 1 type 2 homolog
	2	*TTHA1607*	Thymidylate kinase (dTDP synthesis)
	3	*TTHA1608*	Uncharacterized protein
	4	*TTHA1609*	Uncharacterized protein
Y	1	*TTHA0733*	Transcriptional regulator MarR family
	2	*TTHA0732*	Uncharacterized protein (transporter activity)
	3	*TTHA0731*	Uncharacterized protein (transporter activity)
	4	*TTHA0730*	Uncharacterized protein (secreted)
	5	*TTHA0729*	Probable efflux transporter, AcrB/AcrD/AcrF family
	6	*TTHA0728*	Uncharacterized protein (AB_hydrolase_5 domain)
	7	*TTHA0727*	Uncharacterized protein (peroxiredoxin activity)
~	1	*TTHA0734*	Hemolysin-related protein (transmembrane, FAD-binding, oxidoreductase)
	2	*TTHA0735*	Cytidine deaminase
Y	(3)	*TTHA0706*	Cation-transporting ATPase (transmembrane)
	4	*TTHA0707*	Pyridoxal 5'-phosphate synthase subunit PdxT (vitamin B6 biosynthesis, l-glutamine hydrolysis)
Y	(4)	*TTHA0787*	Uncharacterized protein (38 aa)
	5	*TTHA0786*	Glycerate dehydrogenase/glyoxylate reductase (oxidoreductase)
	6	*TTHA0785*	Uncharacterized protein (transmembrane)
	7	*TTHA0784*	Uncharacterized protein (transmembrane)

(~) Indicates that while a promoter is present, the SbtR-binding site does not overlap core elements (*e*.*g*., –35, –10, +1). (N) Single transcriptional unit, not part of an operon. (3) Number indicates gene position within an operon. Parentheses indicates SbtR site is not before the first gene of an identified operon. Values in italics indicate differences between databases in their identification of operon members.

## Discussion

To better understand the biological functions of orphan transcriptional regulators in model organisms, we sought to develop a general combinatorial approach for determining their DNA binding specificities and then use this information to identify potential genes regulated by these proteins. As a proof of concept, we chose the extreme thermophile *T*. *thermophilus* transcriptional regulator SbtR, an experimentally tractable protein for which some information was already available. This allowed us to validate our approach with a relatively known protein before embarking on less well known putative transcriptional regulators in *T*. *thermophilus* and other organisms.

Previously, Agari *et al*. used four rounds of SELEX with a C-terminal his6-tagged SbtR and PCR-generated inserts from a *T*. *thermophilus* genomic library to identify DNAs containing SbtR binding sites [24]. Of the 46 clones sequenced, 13 contained *bona fide T*. *thermophilus* genomic sequences from 170 to 428 bp in length. Ten of these contained sequences upstream of ORFs, including *TTHA1316* (six clones), *TTHA0787* (two clones), *TTHA0027* (one clone), and *TTHA0760* (one clone). Comparison of these four upstream sequences identified similar (pseudo)palindromic sequences that could constitute a SbtR binding site: (*TTHA1316*) 5′-TGACCGGTCA-3′, (*TTHA0787*) 5′-TGACCCGCGGGTCA-3′, (*TTHA0027*) 5′-TGACCCGCTGGTCA-3′, and (*TTHA0760*) 5′-TGACCCAAATGCCC-3′, leading to a postulated consensus sequence 5′-TGACCCNNKGGTCA-3′, with the predicted binding site underlined. In the studies we describe here, seven rounds of REPSA with wild-type SbtR and a synthetic library of billions of 24-mer sequences yielded position weight matrices shown graphically as a sequence logos (Fig 6). Sequences fitting the 14-mer palindromic matrix, assumed to be the preferred binding site of the homodimeric SbtR protein, were found in 938/1000 inserts, giving an extraordinary statistical significance of E = 1.7 × 10^−2443^. Most important, the SbtR consensus described by Agari *et al*. may be found in the 14-mer consensus we identified by REPSA: 5′-TGACYRNNYRGTCA-3′, especially those nucleotides at the periphery of the sequence (underlined). Thus, the two combinatorial approaches, genomic SELEX and REPSA, identify comparable binding site sequences for SbtR. Notably, REPSA yielded more information regarding potential DNA sequences that may be recognized by SbtR, given the very large number of sequences investigated. Genomic SELEX, on the other hand, gives a better perspective on those naturally occurring sequences that recognize SbtR, which need not be those of highest affinity to be physiologically relevant.

Agari *et al*. performed limited biophysical studies on SbtR [24]. Using both isothermal calorimetry and surface plasmon resonance, they investigated the binding of SbtR to the exemplary binding site found upstream of *TTHA0027*, 5′-TGACCCGCTGGTCA-3′. These provided equilibrium binding constant measures ranging from 63 nM to 5.8 nM under ambient conditions. We used a variety of molecular biological and biophysical techniques, including REPA, EMSA, and BLI, to investigate SbtR binding to a variety of sequences. REPA indicated that 20 nM SbtR could effectively inhibit BpmI cleavage on a selection template containing our consensus sequence 5′-TGACTGGCCAGTCA-3′, while 2 nM could not. Such provides only a rough measure of SbtR binding affinity, as this assay entails a competition between SbtR and the IISRE cleavage domain for access to the same site. EMSA provided a better measure of the equilibrium binding constant for SbtR and our consensus sequence, with a K_D_ ~ 2.5 nM for an initial, faster mobility S_1_ SbtR-DNA complex and K_D_ ~ 16 nM for a slower mobility S_2_ SbtR-DNA complex. Given that SbtR exists as a homodimer in solution and binds a palindromic sequence, we expect the S_1_ complex to have a 2:1 stoichiometry of SbtR to DNA. Therefore, we believe the S_2_ complex to have a 4:1 SbtR:DNA stoichiometry, although we do not know whether both homodimers simultaneously bind DNA. BLI provided us with the most precise data for SbtR binding to DNA, allowing a direct determination of SbtR-DNA binding kinetics in solution. For the consensus sequence, SbtR had an association rate of 498,000 Ms^-1^ and a dissociation rate of 2.1 × 10^−4^ s^-1^ for a calculated K_D_ = 0.42 nM. Notably, this value is lower than what we determined by REPA and EMSA and is more than 10-fold lower than reported previously [24]. The ease of performing BLI assays and the quality of its data prompted us to investigate mutants of the consensus SbtR binding sequence, to determine the importance of each nucleotide in determining the stability of the SbtR-DNA complex. Mutations throughout the palindromic half site affected the equilibrium binding constant from negligible to 33-fold weaker binding, with the greatest changes occurring at the most significant bases and involving changes in dissociation rather than association. Taken together, our biophysical data expands on that obtained previously for SbtR and demonstrates the strengths and weaknesses of different molecular biology/biophysical methods for investigating protein-DNA binding.

Bioinformatic studies were performed by Agari *et al*. to identify additional SbtR binding sites within the *T*. *thermophilus* genome and to identify those genes that may be regulated by SbtR [24]. Six additional SbtR sites beyond those identified through genomic REPSA were identified, two within upstream sequences of *TTHA1330* (5′-TGACCCGTTGGTCA-3′) and *TTHA1821*/*TTHA1822* (5′-TGACCAAAGGGTCA-3′) and the others within ORFs. To validate these genes and those previously identified, *in vitro* run-off transcription assays with *T*. *thermophilus* RNA polymerase were performed. They observed that SbtR could reduce transcription of templates containing upstream sequences from *TTHA0787*, *TTHA0027*, and both *TTHA1821* and *TTHA1822*, suggesting that these promoters are negatively regulated by SbtR *in vivo*. Investigating the operon structure involving these genes, they were able to identify a total of 10 genes that are presumably regulated by SbtR. These included probable transporters, enzymes involved in sugar- or amino acid-metabolism, and nucleic acid-related enzymes, with a role for SbtR in mediating oxidative stress responses being proposed. We, too, performed bioinformatic studies using our consensus sequence. Of the top 14 sites identified by FIMO, nine were located upstream of 12 different genes. These were investigated for the presence of putative promoter sequences. All had identifiable core promoter sequence elements (–35 and –10 elements), with 10 having overlapping sequences with SbtR binding sites. Notably, our top candidates included several genes previously identified, including *TTHA1316*, *TTHA1330*, *TTHA1821*, *TTHA1822*, *TTHA0027*, and *TTHA0787* as well as others not previously identified (*TTHA1315*, *TTHA1605*, *TTHA1606*, *TTHA0733*, *TTHA0734*, and *TTHA0706*). These genes are part of several postulated introns; thus, as many as 44 genes may potentially be regulated by SbtR. Given that the identities of many ORFs in the *T*. *thermophilus* genome remain as yet unknown, it is not surprising that many of the genes potentially regulated by SbtR (15/44) are presently described in the KEGG and UniProtKB databases as encoding uncharacterized proteins [53,54]. However, most (29/44) have some potential function ascribed to their proteins, based primarily on sequence homology to known proteins in other organisms. Strikingly, the majority of these proteins (15/29) are listed as potential membrane efflux proteins, transporters, or channels. Next in abundance (6/29) are several genes encoding potential nucleic acid biosynthetic or modifying proteins, including a nicotinamide mononucleotide deamidase, RNA cyclic phosphodiesterase, endoribonuclease, GTP cyclohydrolase, and thymidylate kinase. Most fascinating is the MarR-family transcriptional regulator encoded by *TTHA0733* that may be a target for SbtR regulation. It is tempting to speculate that this protein may in fact directly or indirectly affect SbtR expression, thereby providing a feedback regulation between these proteins. If true, such would be one of the first steps towards understanding the transcriptional regulatory network present in *T*. *thermophilus*, an ultimate goal of our research.

Taken together, our studies demonstrate that REPSA is an effective means to select for DNAs that specifically and avidly bind a wild-type transcription regulator, even when present in a partially purified fraction. We also found that a simple massively parallel sequencing platform such as the Ion PGM using a 314 Ion chip can be used to obtain the sequences of tens of thousands of selected DNA, more than is needed for determination of a consensus sequence. Sequence analysis using freely available motif-based tools (MEME Suite) can be used to *de novo* discover motifs among these sequences and generate position weighted matrices/sequence logos with exceptionally high statistical significance. Validation of protein binding to a consensus sequence could be obtained using either molecular biology or biophysical techniques, with EMSA being best to define binding stoichiometry and BLI best for obtaining kinetic parameters. Use of point-mutated consensus sequences and BLI allowed us to determine the importance of different bases within the consensus sequence for directing protein-DNA binding affinity, which corresponded well with data from the position weight matrix/sequence logo. Information from the position weight matrix could then be used to identify sequence matches within an organism’s genome, with these being compared with available databases to determine whether they mapped to putative regulatory regions for specific genes and/or operons. Bacterial promoters could then be predicted using different algorithms, and the locations of conserved elements compared with protein binding sites to indicate the possibility of regulation. Finally, the predicted protein products of potentially regulated genes and/or operons may be analyzed with regards to their proposed biological functions, thereby leading to hypotheses as to the biological role of the transcription regulator under investigation. Our studies with the *T*. *thermophilus* transcriptional repressor SbtR allowed us to define its DNA-binding specificity, identify potentially regulated genes within the *T*. *thermophilus* genome, and propose a biological role for this protein. This provides a general framework for future studies on understanding the DNA-binding properties and potential biological functions for orphan transcriptional regulators, both in *T*. *thermophilus* and other organisms.

## Materials and Methods

### Oligonucleotides

Oligonucleotides used in this study were synthesized by Integrated DNA Technologies and purified as per manufacturer’s suggestion. These included single-stranded selection template precursors, PCR primers, and defined SbtR-binding DNA probe precursors. Sequences of these oligonucleotides are provided in Table 1. Oligonucleotides were prepared in water and stored at −20°C. Concentrations were determined using a Thermo Scientific NanoDrop 2000 spectrophotometer and extinction coefficients provided by the manufacturer. The average nucleotide composition of the randomized cassette in REPSA selection template ST2R24 was estimated as 25% A, 25% C, 25% G, and 25% T at each position by direct sequencing of the DNA pool. Double-stranded DNA was prepared from single-stranded oligonucleotides by PCR using New England Biolabs (NEB) *Taq* DNA polymerase and standard reaction conditions as indicated by the manufacturer. REPSA selection template ST2R24 was initially prepared with minimal PCR cycles (6) to ensure that the resulting product was primarily duplex DNA with fully annealed randomized cassette regions.

### Expression and purification of SbtR protein

Plasmid pET-sbtR, which contains the *T*. *thermophilus TTHA0167* (*sbtR*) gene under the control of a T7 promoter in the *E*. *coli* expression vector pET-11a, was obtained from the RIKEN Structural Biology Laboratory and was the generous gift of Dr. Akeo Shinkai [24]. Competent *E coli* strain BL21(DE3) bacteria transformed with pET-sbtR were propagated in 50 ml LB medium supplemented with 100 μg/ml ampicillin at 37°C/300 rpm until an OD_600_ of 0.7 was obtained. Isopropyl β-D-1-thiogalactopyranoside (IPTG) to a final concentration of 1 mM was then added, and incubation continued for an additional 5 h. Bacteria were pelleted by centrifugation (4000 × *g*, 10 min, 4°C) and resuspended in 0.5 ml cold 40 mM Tris-Cl (pH 7.7) and 200 mM NaCl. Bacteria were lysed by five cycles of sonication (3 W, 20 s on/20s off, 0°C) with notable clarification of bacterial suspensions being observed. Bacterial debris was pelleted following centrifugation (21,000 × *g*, 10 min, 4°C) and the soluble fraction recovered by decantation. This was heated at 70°C for 10 min to denature *E*. *coli* proteins, with substantial clouding being observed. These insoluble proteins were then pelleted by centrifugation (21,000 × *g*, 10 min, 4°C). The resulting supernatant was then diluted with an equal volume of glycerol and mixed by gentle rocking for 30 min at 4°C until homogeneous. Stock SbtR was stored at −20°C. Protein concentration was determined using a Bio-Rad protein assay and estimated at 4.36 mg/ml. Protein purity was determined by SDS-PAGE and estimated as greater than 90% pure (Fig 2, lane 3). Given that SbtR is an 189 amino acid TetR/AcrR family transcription regulator protein with a molecular mass of 21,539 Da (UniProtKB, http://www.uniprot.org/uniprot/Q5SLX6), this corresponds to our stock solution being no more than 200 μM SbtR monomer or 100 μM SbtR_2_, the dimeric form presumed to bind DNA.

### REPSA selection

For our REPSA selections with SbtR, 2 ng (42 fmole) pool DNA was incubated in a 20 μl volume containing 50 mM K acetate, 20 mM Tris acetate, 10 mM Mg acetate and 2 μg BSA (NEB CutSmart buffer, pH 7.9 @ 25°C) with 36 nM purified SbtR (18 nM SbtR dimer) as indicated, for 20 min at 55°C to affect binding. Afterward, samples were equilibrated at 37°C for 5 min before addition of 0.2 U IISRE, either NEB FokI or BpmI, as indicated. Incubation at 37°C was continued for an additional 5 min to affect cleavage, with reactions terminated by placing samples on ice. For PCR amplification of REPSA selected DNA, 2 μl of the selection reaction was added to each of three 23 μl PCR reactions containing 1× NEB Standard *Taq* Reaction Buffer, 200 μM dNTPs, 100 nM each primers ST2L and IRD7_ST2R, and 0.625 U NEB *Taq* DNA polymerase assembled on ice. Thermocycling conditions used for PCR amplification were 30 s denaturation at 95°C, 30 s annealing at 56°C, and 60 s elongation at 72°C. For each round of REPSA, 6, 9, and 12 cycle PCRs were performed to obtain maximal amplification of selected DNA while maintaining their double-stranded integrity. Quantitation of PCR products was obtained using a Thermo Fisher Qubit ds DNA HS assay. Native 10% PAGE analysis was performed on 2 μl aliquots of each sample (DNA control, cleavage control, REPSA selection, PCR amplification 6 cycle, 9 cycle, 12 cycle) to which 2 μl NEB Orange Gel Loading Dye supplemented with 1% SDS was added. Visualization of IRD7-labeled DNA species was performed using a LI-COR Odyssey Imager.

### Electrophoretic Mobility Shift Assays

Two types of electrophoretic mobility shift assays (EMSA) were performed in this study: those with mixed DNAs from REPSA selections and those with defined DNAs. In the former, 5 μl reaction volumes containing 1 ng (21 fmole) PCR-amplified DNA from the indicated round of REPSA selection, 1× NEB CutSmart buffer, and the indicated concentration of SbtR were incubated for 20 min at 55°C to affect DNA binding. Afterward, 2 μl 20% glucose containing 0.9% Orange G dye was added and the samples loaded onto a ½× Tris-borate-EDTA, 10% polyacrylamide gel before electrophoresis at 10 V/cm for 60 min. IR-dye labeled species were visualized using a LI-COR Odyssey Imager. For EMSAs with defined DNAs, reactions were performed as above except that the DNAs present include 1 nM each 63-bp IRD7-labeled SbtR consensus DNA (red) and 86-bp IRD8-labeled REPSAis control DNA (green).

### Restriction Endonuclease Protection Assays

Other than the restriction endonuclease protection assays (REPA) performed as part of a round of REPSA, REPAs were also performed with defined DNAs to ascertain their protein-DNA binding characteristics. Defined DNA REPAs contained 1 nM each 63-bp IRD7-labeled SbtR consensus DNA (red) and 86-bp IR8-labeled REPSAis control DNA (green) in a 5 μl reaction volume together with 1× NEB CutSmart buffer and the indicated final concentration of SbtR. These were incubated at 55°C for 20 min to affect binding, followed by equilibration at 37°C for 5 min. IISRE (0.2 U BpmI) was then added and cleavage allowed to proceed at 37°C for 5 min before stopping on ice. NEB Orange Gel Loading Dye supplemented with 1% SDS (2 μl) was added and the samples analyzed by native 10% PAGE analysis as described above. Visualization of IR-dye labeled DNA species was performed using a LI-COR Odyssey Imager.

### Biolayer Interferometry

SbtR binding kinetics and dissociation constant were determined using biolayer interferometry (BLI) with a Pall Life Sciences fortéBIO Octet^QK^ instrument. Biotinylated dsDNA probes containing either the 14-bp consensus SbtR binding sequence (wt) or point-mutated sequences (m1 –m7) were synthesized by standard PCR using ST2L and Bio_ST2R primers and ST2_SbtR_R7_ templates indicated in Table 1. Streptavidin Dip and Read Biosensors (fortéBIO) were initially hydrated in Buffer BLI containing 20 mM Tris-Cl (pH 7.7 @ 25°C), 100 mM NaCl, 1 mM EDTA, and 0.05% Tween 20 for 5 min at 30°C. Sensors were then immersed in BLI buffer containing 1.4 nM biotinylated DNA probe for 15 min to affect maximal binding to the sensor. After washing for 5 min in BLI buffer, a baseline was determined and binding reactions initiated. Sensors were incubated with different concentrations of SbtR dimer protein (1.85, 5.56, 16.7, 50, 150 nM) for 5 min to permit association, followed by washing in BLI buffer for 15 min to permit dissociation. BLI readings were taken at 1.6 s intervals throughout these incubations. Data from the association and dissociation steps were imported into GraphPad Prism 5.03 and analyzed using their single-state *Association then Dissociation* user-defined equation with a least square (ordinary) fit and the breakpoint set at 302.4 s. Nonlinear fit results are shown graphically together with the experimentally derived data points. Global (shared) values from these analyses, including *k*_on_ and *k*_off_ rate constants, K_D_ equilibrium binding constant, and R^2^ goodness-of-fit determinations are provided in Table 2.

### Massively Parallel Sequencing of REPSA-selected DNAs

REPSA-selected DNAs were massively parallel sequenced using a Thermo Fisher Ion Personal Genome Machine (Ion PGM) and its semiconductor sequencing technology. Protocols essentially followed those provided by the manufacturer.

Amplicon libraries were prepared using fusion primers containing Ion PGM A or trP1 sequences fused to our ST2R or ST2L sequences, respectively. Please see Table 1 for the list of PCR primers used and their Publication 4468326, Revision C for overall scheme. After limited PCR amplification under our standard PCR conditions (6 cycles, 95°C/30 s, 54°C/30 s, 72°C/60 s), DNA pools were isolated from this reaction and used to seed standard PCR reactions (30 cycle) with A_uni and trP1_uni primers. Resulting DNAs were analyzed by native 10% PAGE and ethidium bromide staining. Each was found to yield a tight band at the expected length (139 bp).

Ion PGM individual sequencing particles (ISPs) were prepared using an Ion PGM Template OT2 200 kit and an Ion OneTouch 2 instrument following manufacturer’s instructions (Publication MAN0007220, revision A.0). Input DNA was typically pools of several experiments (4–10), barcoded to allow their identification. Template-positive ISPs were enriched using an Ion OneTouch ES. Quality control was performed using an Ion Sphere Quality Control assay and Thermo Fisher Qubit 2.0 Fluorimeter with v3.10 firmware. Values were on the high end of acceptability (38), suggesting some polyclonal ISPs in our preparations. Massively parallel sequencing was performed on the Ion PGM using an Ion PGM Sequencing 200 kit v2 and Ion 314 chip v2 following manufacturer’s instructions (Publication MAN0007273, revision 3.0, 07Aug2103) with the exception that sequencing chips were loaded using a simplified chip loading protocol (Publication MAN0007517, Revision 1.0). Sequencing was performed for 500 flows and data transferred to the Ion Torrent server for processing. Sequencing run summary was 1.26M wells addressable, 870k ISP loaded, and 418k high-quality, clonal ISPs in the final sequence library. Each barcoded species yielded 1.9–3.4M bases ≥Q20 with mean read lengths 49–53 bp. These data were outputted as separate fastq files for further processing.

### Bioinformatics

Sequence data in fastq format was first processed using a script provided by Ying Xie (Computer Science, Kennesaw State University). This script (Sequence1.java) takes information from an accompanying file (Parameter.txt) to identify those sequences that have intact ST2R/ST2L flanks and a proper insert length of 24 bases. It then strips extraneous information (ST2R/ST2L flanking sequences, quality value information), rendering the sequences in a format amenable for further analysis. Identical and unique sequences were identified in this file using DuplicatesFinder v1.1 (http://proline.bic.nus.edu.sg/~asif/tools/DuplicateFinder.zip). Bioinformatic analysis of REPSA sequences was performed using the MEME Suite of software (v4.10.2) via their website (http://meme-suite.org/) [45]. Multiple Em for Motif Elicitation (MEME) was used to generate sequence logos for the top three motifs identified in a population of 1000 sequences, the limit for this software. Default options were used except for the restriction to palindromes, where indicated. Top motifs were then submitted to Find Individual Motif Occurrences (FIMO) to identify their best matches within the *T*. *thermophilus* HB8 genome. The top matched sequences identified by FIMO were then investigated in the context of their genomic sequences. Sequences ±300 bp of the FIMO matched sequence were analyzed online by both Softberry BPROM (http://www.softberry.com/) and University of Groningen Genome2D (http://genome2d.molgenrug.nl/) to identify potential promoter elements [49,50]. Operons were identified using databases at the University of Georgia (DOOR^2^, http://csbl.bmb.uga.edu/DOOR/) and the Universidad Nacional Autónoma de México (ProOpDB, http://operons.ibt.unam.mx/OperonPredictor/) [51,52]. Information on identified/postulated protein functions for potential SbtR-regulated genes was obtained from both the KEGG Genome database (*Thermus thermophilus* HB8) and the UniProt Knowledgebase database (*Thermus thermophilus* strain HB8 / ATCC 27634 / DSM 579) [53,54].
